# The predictive value of combining CAAP-AF score and epicardial adipose tissue thickness for early recurrence after catheter ablation in atrial fibrillation patients

**DOI:** 10.3389/fcvm.2026.1839244

**Published:** 2026-05-20

**Authors:** Liqing Lin, Shuxuan Huang, Linfang Ke, Jinxin Lan, Ming Chen

**Affiliations:** Department of Ultrasound, Zhangzhou Affiliated Hospital of Fujian Medical University, Zhangzhou, China

**Keywords:** atrial fibrillation, CAAP-AF score, catheter ablation, echocadiography, epicardial adipose tissue

## Abstract

**Background:**

Atrial fibrillation (AF) is the most common arrhythmia globally, associated with a significant burden of stroke, heart failure, and reduced quality of life. Early recurrence after catheter ablation for AF remains a common and challenging issue.

**Objective:**

Our aim was to investigate the correlation between epicardial adipose tissue (EAT) thickness and early recurrence after catheter ablation in patients with AF, and to evaluate the incremental predictive value of EAT thickness when combined with the CAAP-AF score.

**Methods:**

A total of 56 AF patients who underwent catheter ablation between March 2023 and November 2023 at Zhangzhou Affiliated Hospital of Fujian Medical University were included in this study. The CAAP-AF score was calculated, and EAT thickness was measured for each patient. Patients were divided into two groups based on follow-up outcomes: early recurrence and no recurrence. Receiver operating characteristic (ROC) curve analysis was performed to evaluate the predictive value of the CAAP-AF score, EAT thickness, and their combination for early recurrence after catheter ablation.

**Results:**

Early recurrence occurred in 13 patients, while 43 patients had no recurrence. Multivariate logistic regression analysis demonstrated that both the CAAP-AF score (OR = 2.10, 95% CI: 1.09–4.05, *p* = 0.027, *β*=0.74) and EAT thickness (OR = 3.71, 95% CI: 1.33–10.34, *p* = 0.012, *β*=1.31) were independent predictors of early recurrence. The AUC values for the CAAP-AF score, EAT thickness, and their combination in predicting early recurrence were 0.871, 0.800, and 0.914, respectively. Furthermore, intra-class correlation coefficients (ICC) and Bland-Altman analysis demonstrated excellent reproducibility of EAT thickness measurements via ultrasound.

**Conclusion:**

Both the CAAP-AF score and EAT thickness are independent risk factors for early recurrence after catheter ablation in AF patients. Combining these two parameters enhances the predictive accuracy for early recurrence.

## 1 Introduction

Atrial fibrillation (AF) is the most prevalent cardiac arrhythmia globally (1), characterized by rapid and disorganized atrial electrical activity. This condition not only increases the risk of stroke and heart failure but also diminishes the quality of life of affected individuals. With the aging of the population, the prevalence of AF is expected to rise, further straining healthcare resources. Catheter ablation has been established as a first-line rhythm control strategy for AF in clinical guidelines (2), offering a therapeutic option for patients who remain symptomatic despite medical therapy. However, the high recurrence rate following ablation has emerged as a critical issue warranting clinical attention (3).

The recurrence of AF after catheter ablation is a complex phenomenon influenced by a multitude of factors, including clinical characteristics, anatomical variations, and biological factors. Identifying patients at high risk of early recurrence is crucial for tailoring post-ablation management strategies, such as intensified monitoring or the use of adjunctive antiarrhythmic therapies. A recent meta-analysis suggested that epicardial adipose tissue (EAT) thickness is closely associated with an increased risk of AF recurrence post-ablation, making it a potential biomarker of interest (4). EAT, a metabolically active fat depot surrounding the heart, has been shown to secrete various pro-inflammatory cytokines and adipokines, which contribute to atrial fibrosis, inflammation, and electrical remodeling, thereby increasing the likelihood of AF recurrence (5).

Among the risk scoring systems developed to predict post-ablation recurrence, the CAAP-AF score has demonstrated strong predictive performance (6). This score incorporates clinical and anatomical parameters, such as coronary artery disease, left atrial diameter, age, AF type, antiarrhythmic drug failure, and gender, providing a practical tool for assessing the risk of recurrence. However, these clinical scores do not account for biological factors, such as EAT, which may play a significant role in post-ablation recurrence. This study aims to investigate the incremental predictive value of EAT thickness when combined with the CAAP-AF score for assessing recurrence risk in AF patients undergoing catheter ablation.

The integration of EAT thickness with the CAAP-AF score could potentially enhance the predictive accuracy of recurrence risk models, allowing for more personalized and effective clinical decision-making. By improving the identification of patients at high risk for early recurrence, clinicians can optimize post-ablation management strategies, potentially reducing the burden of early AF recurrence and improving long-term patient outcomes.

This study, therefore, seeks to address an important gap in the current literature by combining a well-established clinical score with a novel biological marker. We hypothesize that the addition of EAT thickness to the CAAP-AF score will significantly improve the predictive accuracy of the model, providing a more comprehensive assessment of the individual risk profile for AF recurrence. The findings of this study could have significant implications for clinical practice, guiding the development of more tailored therapeutic approaches and improving the long-term outcomes for patients with AF.

## 2 Materials and methods

### 2.1 Study population

This bidirectional cohort study prospectively included 72 AF patients who presented at our hospital for planned catheter ablation between March 2023 and November 2023. Sixteen patients were excluded (six patients aged >80 years and ten who did not undergo the procedure for various reasons), resulting in a final cohort of 56 patients.

This study protocol was reviewed and approved by the Ethics Committee of Zhangzhou Affiliated Hospital of Fujian Medical University (Approval Code: 2024KYZ407). Written informed consent was obtained from all participants prior to enrollment. The consent process included detailed explanations of: (i) Research objectives and catheter ablation procedures. (ii) Potential risks (e.g., procedural complications) and benefits. (iii) Data anonymization protocols: All echocardiographic and clinical records were de-identified prior to analysis. (iv) Voluntary participation with guaranteed withdrawal rights at any stage.

Original signed consent forms are securely archived at the principal investigator’s institution for a minimum of 5 years post-study completion in accordance with China’s Regulations on Human Genetic Resources Management.

Inclusion criteria: (i) Patients undergoing their first catheter ablation for atrial fibrillation. (ii) Complete pre-procedural echocardiographic data available. (iii) Absence of left atrial appendage thrombus confirmed by preoperative transesophageal echocardiography or left atrial pulmonary vein imaging.

Exclusion criteria: (i) Age <20 years or >80 years. (ii) Rheumatic or other valvular heart diseases. (iii) Hyperthyroidism. (iv) Active connective tissue diseases. (v) Loss to follow-up during the study period.

### 2.2 Clinical data collection and risk scoring

Clinical data, including demographic information, comorbidities, disease duration, AF type, and biochemical parameters, were collected from the hospital’s electronic medical record system.

The CAAP-AF score (7), ranging from 0 to 13, was calculated for each patient based on the following criteria: (i) Coronary artery disease: 1 point. (ii) Left atrial diameter (LAD): <4.0 cm (0 points), 4.0–4.4 cm (1 point), 4.5–4.9 cm (2 points), 5.0–5.4 cm (3 points), ≥5.5 cm (4 points). (iii) Age: < 50 years (0 points), 50–59 years (1 point), 60–69 years (2 points), ≥70 years (3 points). (iv) Persistent AF: 2 points. (v) Number of failed antiarrhythmic drugs: 0 (0 points), 1–2 (1 point), >2 (2 points). (vi) Female gender: 1 point.

### 2.3 Echocardiographic image acquisition

Echocardiographic imaging was performed using a GE Vivid E95 Color Doppler Ultrasound System (GE Healthcare, USA). Transthoracic echocardiography (TTE) employed an M5Sc probe (frequency: 1.7–3.3 MHz), while transesophageal echocardiography (TEE) utilized a 6Tc-RS probe (frequency: 2.9–8.0 MHz). TEE was used to examine the left atrial appendage at multiple levels to rule out thrombus formation.

Patients were positioned in the left lateral decubitus position and asked to breathe normally. For TTE, the left atrial anteroposterior diameter was measured in the parasternal long-axis view, with the aortic annulus serving as the reference.

EAT thickness was measured following the American Society of Echocardiography (ASE) and European Association of Cardiovascular Imaging (EACVI) guidelines. All measurements were acquired in the parasternal long-axis view at end-systole (aortic valve closure phase), focusing on the interface between the right ventricular free wall and the visceral pericardium. The EAT thickness was defined as the hypoechoic space located 1 cm apical to the aortic valve plane and measured using the inner-edge to inner-edge technique, consistent with ASE standards for vascular wall quantification. Three consecutive cardiac cycles were analyzed, and the mean value was recorded. Examinations with suboptimal acoustic windows (defined as >50% unclear visualization of the RV-pericardium interface) or pericardial thickening (>2 mm) were excluded from analysis. All measurements were performed by a board-certified cardiologist with over 5 years of echocardiography experience, who remained blinded to clinical outcomes throughout the study. Intra- and inter-observer reproducibility were rigorously evaluated using intra-class correlation coefficients and Bland-Altman analysis, as detailed in the Results section.

**Figure 1:**
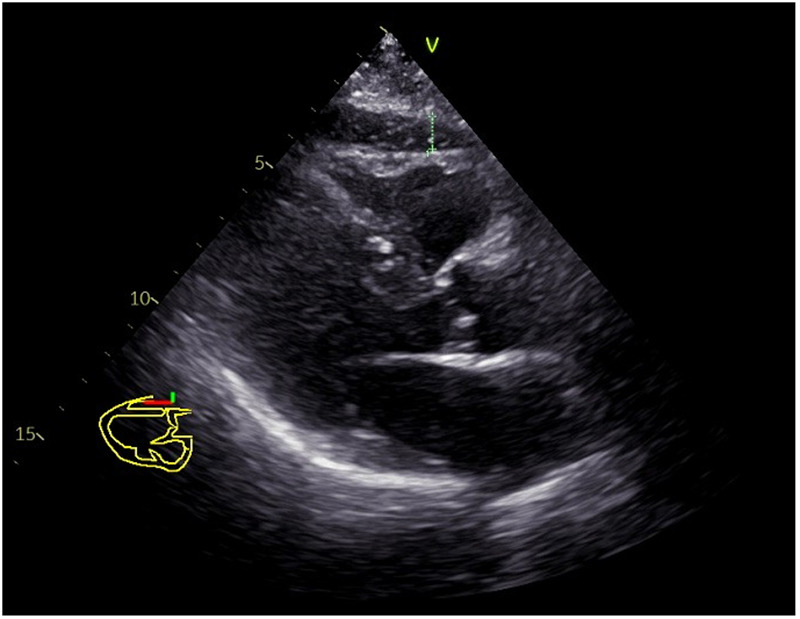
Transthoracic ultrasound measurement of EAT.

EAT thickness was measured at end-systole in the parasternal long-axis view, defined as the relatively hypoechoic area between the right ventricular free wall and the visceral pericardium (Figure 1). Measurements were performed over three consecutive cardiac cycles, and the mean value was used for analysis.

All echocardiographic images were independently obtained by a senior attending physician with extensive experience. Dynamic images were stored on the ultrasound system for subsequent analysis. To assess inter-observer reproducibility, a randomly selected subset of 20 cases was reanalyzed by a trained junior resident who was blinded to the original measurements. Intra-observer reproducibility was evaluated by having the same operator reanalyze the images one week later to eliminate variability introduced by either the patient or the examiner. These measures ensured the accuracy and reliability of the study results.

### 2.4 Catheter ablation record

All patients underwent radiofrequency catheter ablation (RFA) under the guidance of a three-dimensional electroanatomical mapping system (CARTO®, Biosense Webster, Irvine, CA, USA; or EnSite™, Abbott, St. Paul, MN, USA), selected according to system availability and operator preference. Pulmonary vein isolation (PVI) was the primary lesion set, performed as circumferential ablation encircling the left and right-sided pulmonary veins. Procedural endpoints were bidirectional PVI with confirmation of both entrance and exit block: entrance block was defined as abolition of pulmonary vein potentials or electrical dissociation between the left atrium and pulmonary veins; exit block was defined as failure to capture the left atrium during high-output pacing from within the pulmonary veins. In selected patients with persistent atrial fibrillation, additional linear ablation (e.g., roof line and/or mitral isthmus line) was performed at the operator’s discretion. Procedural success was defined as confirmed entrance and exit block in all four pulmonary veins. All antiarrhythmic drugs were discontinued for at least five half-lives prior to the procedure. All procedures were performed by two experienced operators.

### 2.5 Postoperative follow-Up and definition of early recurrence

Early recurrence (8): Early recurrence of atrial arrhythmias was defined as the occurrence of atrial fibrillation (AF), atrial flutter, or atrial tachycardia lasting more than 30 s within the first three months following ablation, as documented by a 12-lead electrocardiogram (ECG) or 24-hour Holter monitoring.

Postoperative follow-up was conducted through regular outpatient visits and telephone consultations. ECG evaluations were performed at one month and three months post-ablation. Patients presenting with symptoms related to AF, such as palpitations, chest tightness, or dizziness, or symptoms resembling their pre-ablation AF episodes, were instructed to undergo immediate ECG or 24-hour Holter monitoring.

The follow-up endpoint was defined as early recurrence of AF. Patients who did not experience recurrence were followed up until the end of the three-month postoperative period.

### 2.6 Statistical analysis

Statistical analyses were performed using SPSS version 27.0, R version 4.3.0, and GraphPad Prism 9. Patients were divided into two groups based on their follow-up outcomes: the early recurrence group and the non-recurrence group.

#### 2.6.1 Data presentation

Continuous variables were tested for normality using the Shapiro–Wilk test. Data following a normal distribution were expressed as mean ± standard deviation (*χ* ± s), while non-normally distributed data were presented as median (P25, P75). Categorical variables were described as frequencies and percentages.

#### 2.6.2 Comparative analyses

Independent sample t-tests were used to compare continuous variables between the two groups. Categorical variables were compared using the chi-square test. All hypothesis tests were two-tailed, and a *p*-value <0.05 was considered statistically significant.

#### 2.6.3 Univariate and multivariate analyses

Univariate analysis was conducted to identify potential factors associated with early recurrence. Variables with *p* < 0.05 were further examined for collinearity. Factors with strong correlations and no multicollinearity were retained for multivariate logistic regression analysis to determine independent predictors of early recurrence after catheter ablation in AF patients. A predictive model was constructed based on the identified factors and visualized as a nomogram.

#### 2.6.4 Diagnostic performance

Receiver operating characteristic (ROC) curves were plotted for each parameter to assess their ability to predict early recurrence, and the area under the curve (AUC) was calculated for each parameter to compare diagnostic performance.

#### 2.6.5 Reproducibility analysis

The reproducibility of EAT thickness measurements was evaluated using intra-class correlation coefficients (ICC) and Bland-Altman plots to assess inter-observer and intra-observer variability. These analyses ensured the reliability of the EAT thickness measurements.

## 3 Results

### 3.1 Baseline characteristics of study participants

Among the 56 patients included in the study, 13 experienced early recurrence, while 43 did not. The baseline characteristics of the study participants are summarized in Table 1.

**Table 1:** Clinicopathologic characteristics of patients with atrial fibrillation.

Variable	No recurrence in the early stages (*n* = 43)	Early recurrence group (*n* = 13)	*χ²*/*t*	*p*
Age (y)	62.00 ± 9.01	68.38 ± 9.99	−2.18	0.033
BMI (kg/m^2^)	25.57 (23.50, 27.34)	24.51 (23.03, 26.17)	−1.12	0.264
Gender (%)			0.99	0.319
Female	15 (34.88)	2 (15.38)		
Male	28 (65.12)	11 (84.62)		
AF Duration (Month)	4.00 (1.00, 12.00)	48.00 (6.00, 108.00)	−2.35	0.019
Types of atrial fibrillation (%)			12.12	<0.001
Persistence	16 (37.21)	12 (92.31)		
Paroxysmal	27 (62.79)	1 (7.69)		
CAAP-AF	3.00 (1.50, 4.00)	6.00 (5.00, 7.00)	−4.06	<0.001
NT-proBNP (ng/L)	372.84 (124.77, 576.67)	780.93 (285.82, 3164.00)	−2.01	0.045
Uric acid (umol/L)	403.00 (327.60, 429.85)	398.10 (336.60, 463.60)	−0.65	0.516
EAT thickness (mm)	4.57 (4.06, 5.47)	5.93 (5.57, 6.61)	−3.24	0.001
LAD（mm）	35.53 ± 6.06	41.31 ± 9.02	−2.67	0.010
Heart rate	78.00 (69.50, 123.00)	86.00 (72.00, 105.00)	−0.53	0.593
Heart rate variability(ms)	124.00 (99.50, 152.00)	162.00 (88.00, 211.00)	−1.26	0.207
Comorbidities				
Hypertension (%)			0.74	0.389
No	24 (55.81)	9 (69.23)		
Yes	19 (44.19)	4 (30.77)		
Diabetes (%)			0.30	0.582
No	35 (81.40)	9 (69.23)		
Yes	8 (18.60)	4 (30.77)		

EAT, epicardial adipose tissue; LAD, left atrial anteroposterior diameter.

### 3.2 Univariate and multivariate regression analyses

Univariate and multivariate regression analysis are shown in Table 2.

**Table 2:** Univariate and multivariate analysis of early relapse.

	Univariate analysis			Multivariate analysis		
	*β*	*p*	OR (95%CI)	β	*p*	OR (95%CI)
Age	0.08	0.042	1.09 (1.01∼1.18)	-	-	-
BMI	−0.09	0.397	0.91 (0.73∼1.13)	-	-	-
Female	1.08	0.194	2.95 (0.58∼15.07)	-	-	-
Course of atrial fibrillation	0.02	0.007	1.02 (1.01∼1.04)	0.03	0.096	1.03 (1.00∼1.06)
Paroxysmal atrial fibrillation	−3.01	0.006	0.05 (0.01∼0.42)	-	-	-
CAAP-AF	0.94	<0.001	2.57 (1.47∼4.49)	0.74	0.027	2.10 (1.09∼4.05)
NT-proBNP	0.01	0.034	1.01 (1.01∼1.01)	-	-	-
Uric acid	0.00	0.437	1.00 (1.00∼1.01)	-	-	-
EAT thickness	1.02	0.003	2.78 (1.41∼5.48)	1.31	0.012	3.71 (1.33∼10.34)
LAD	0.12	0.018	1.12 (1.02∼1.24)	-	-	-
Heart rate	0.00	0.670	1.00 (0.99∼1.02)	-	-	-
Heart rate variability	0.01	0.214	1.01 (1.00∼1.02)	-	-	-
Hypertension	−0.58	0.392	0.56 (0.15∼2.11)	-	-	-
Diabetes	0.66	0.354	1.94 (0.48∼7.93)	-	-	-

OR, odds ratio; CI, confidence interval. CAAP-AF score was collinearity with age and LAD, but the association strength of CAAP-AF score was higher than that of age and LAD (0.94 vs 0.08, 0.12), so only CAAP-AF score was included in the multiple regression model.

Univariate analysis revealed that age, AF duration, AF type, CAAP-AF score, NT-proBNP, left atrial anteroposterior diameter, and EAT thickness were significantly associated with early recurrence after catheter ablation (*p* < 0.05).

Multivariate logistic regression analysis identified CAAP-AF score and EAT thickness as independent predictors of early recurrence (*p* < 0.05).

### 3.3 Development of a predictive model for early recurrence after catheter ablation

A predictive model for early recurrence was developed based on the results of the multivariate regression analysis. The logistic regression equation was:

Logit(P)=−11.14+1.04×EATthickness+0.94×CAAP_AFscore

**Figure 2:**
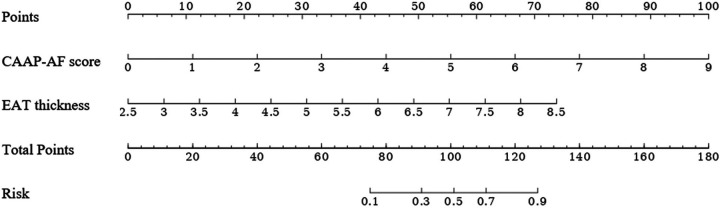
Nomogram based on independent predictors.

A nomogram was generated using R software (Figure 2). The bootstrap resampling method was applied to validate the predictive model. The regression coefficients for CAAP-AF score and EAT thickness were 0.94 (95% CI: 0.41–2.98) and 1.04 (95% CI: 0.40–3.59), respectively.

**Figure 3:**
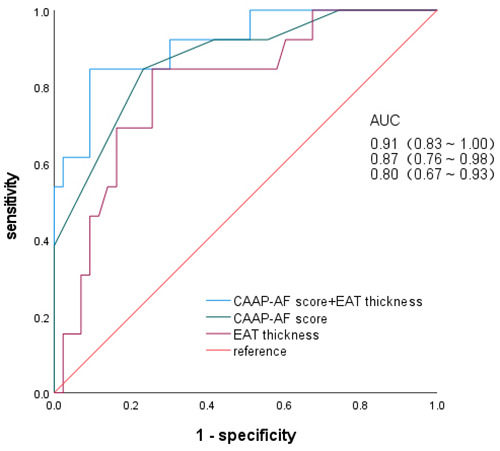
ROC curves of independent predictors and nomogram models.

The areas under the ROC curve (AUCs) for CAAP-AF score, EAT thickness, and their combination in predicting early recurrence were 0.87 (95% CI: 0.76–0.98), 0.80 (95% CI: 0.67–0.93), and 0.91 (95% CI: 0.83–1.00), respectively (Figure 3). The optimal cutoff point, determined by the maximum Youden index (0.753), was 0.322. At this cutoff, the theoretical sensitivity, specificity, positive predictive value, negative predictive value, and overall accuracy of the model were 84.6%, 90.7%, 73.3%, 95.1%, and 89.2%, respectively.

Patients with a predicted risk probability greater than 0.322 were considered to have a high likelihood of early recurrence after catheter ablation.

### 3.4 Reproducibility analysis of EAT thickness

The inter-observer reproducibility of EAT thickness measurements, assessed by two physicians, showed an intraclass correlation coefficient (ICC) of 0.875 (95% CI: 0.690–0.950). For intra-observer reproducibility, when one physician repeated the measurements one week later, the ICC was 0.913 (95% CI: 0.783–0.965), indicating excellent correlation.

**Figure 4:**
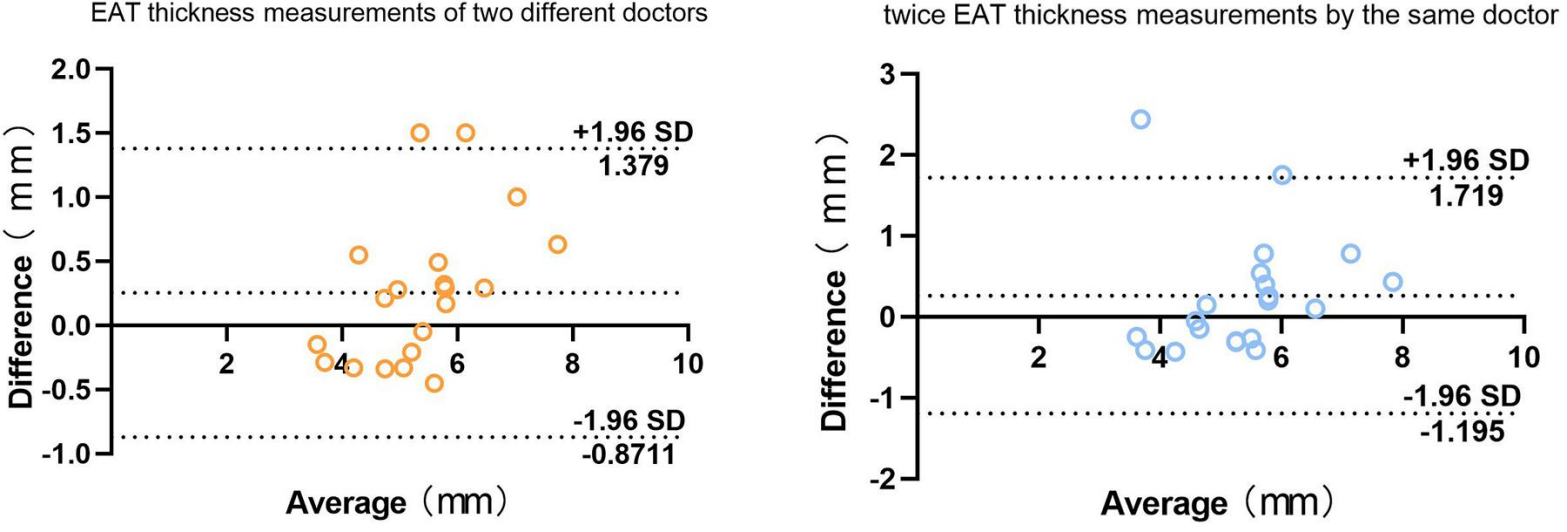
Bland-Altman difference analysis of two physicians and two measurements of EAT thickness by the same physician.

The Bland-Altman analysis demonstrated that the majority of measurement differences, both between observers and for repeated measurements by the same observer, fell within the 95% confidence interval (Figure 4). This result confirmed a high level of agreement, suggesting that EAT thickness measured via ultrasound has excellent consistency and reliability.

## 4 Discussion

Over the past two decades, the global prevalence of atrial fibrillation (AF) has increased by 33%, affecting approximately 37.57 million individuals, which constitutes 0.51% of the global population. Treatment strategies for AF primarily include rate control and rhythm control. The 2023 ACC/AHA/ACCP/HRS Guideline for the Diagnosis and Management of Atrial Fibrillation emphasizes catheter ablation as a superior approach compared to pharmacological therapy for rhythm control, upgrading its recommendation from Class IIa in the 2014 version to Class I (9).

Post-ablation recurrence remains a common challenge. Several risk scoring systems have been developed to predict recurrence, among which the CAAP-AF score stands out due to its simplicity and relatively superior predictive accuracy (AUC: 0.65) compared to other scores (10). The Chinese guidelines 8 designate the first three months post-ablation as a “blanking period,” during which recurrence is not considered a failure of the procedure. However, increasing evidence indicates that early recurrence is associated with a higher risk of late recurrence (11, 12). The BASE-AF2 and MB-LATER risk scores include early recurrence as a significant risk factor. Steinberg et al. reported early recurrence rates ranging from 16% to 65% (12). In this study, the early recurrence rate was 23.2%, consistent with previous findings.

The predictive performance of the CAAP-AF score for early recurrence in this study, with an AUC of 0.87, exceeded its previously reported efficacy in predicting long-term recurrence (13, 14). A CAAP-AF score ≥5 was effective in predicting early recurrence, in agreement with the findings of Sanhoury et al. (15). However, the CAAP-AF score has limitations as it does not incorporate pathological processes, such as left atrial fibrosis, which are closely associated with AF recurrence.

Epicardial adipose tissue (EAT) is a beige adipose tissue located between the visceral pericardium and the myocardium, covering nearly 80% of the heart’s surface (16). It is rich in ganglia and possesses endocrine and paracrine functions, playing a role in AF recurrence following catheter ablation (17). A fundamental study by Japanese researchers demonstrated the relationship between EAT and atrial fibrosis using histological analysis of human left atrial appendage tissue. They concluded that EAT secretes pro-inflammatory cytokines that promote diffuse atrial fibrosis (18).

This study identified EAT thickness as an independent risk factor for early recurrence, consistent with the findings of Leiyu Feng et al. (19). Combining EAT thickness with the CAAP-AF score resulted in a new predictive model with a sensitivity of 84.6%. Bootstrap-based resampling validated the model, confirming that all regression coefficients fell within their respective 95% confidence intervals, indicating the model’s robustness and reliability. Patients with a preoperative predicted recurrence risk probability >0.322 should carefully weigh the risks and benefits of catheter ablation before proceeding with treatment. For such high-risk patients, healthcare providers should focus on intensive preoperative counseling, close postoperative monitoring, and optimization of medical therapy to improve long-term outcomes and quality of life.

It is important to emphasize that our nomogram is not intended to override the established concept of the 3-month blanking period. Rather, it may facilitate individualized post-ablation care by identifying patients who may benefit from closer rhythm surveillance and supportive management. For patients classified as high risk, this may include earlier and more frequent follow-up with additional ECG/Holter monitoring (or device interrogation, when applicable), a more proactive approach to initiating or continuing antiarrhythmic drugs during the blanking period when clinically appropriate, and intensified management of modifiable comorbidities and triggers. Importantly, a high-risk score should not be interpreted as an indication for routine early repeat ablation.

Recent cardiac CT studies suggest that EAT assessment beyond simple thickness may improve risk stratification for AF recurrence after PVI, particularly through peri-atrial/left atrial EAT (LA-EAT) volume and CT attenuation. A recent meta-analysis showed that patients with post-ablation recurrence had significantly higher (less negative) LA-EAT attenuation, supporting its value as a marker of peri-atrial inflammatory remodeling (20). CT studies have also linked greater total or peri-atrial EAT volume to recurrence, with some identifying total EAT volume and LA-EAT attenuation as independent predictors (19, 21). In addition, radiomics models based on LA-EAT features from noncontrast ECG-gated cardiac CT have shown good accuracy in predicting AF (22). In contrast to CT, our study used a simple 2D echocardiographic measurement of EAT thickness, a widely available method that showed good reproducibility in our cohort. Although it cannot provide depot-specific volumetric or tissue characterization of the fat surrounding the left atrium, EAT thickness remained an independent predictor of early recurrence in our cohort. Moreover, when combined with the CAAP-AF score, it improved discriminative performance, suggesting that this practical echocardiographic marker can still offer clinically meaningful incremental value, especially when rapid bedside assessment is needed and CT is unavailable or not routinely performed.

EAT, as a simple and easily measurable biomarker, complements the limitations of the CAAP-AF score. Preoperative EAT thickness measurement via ultrasound, combined with the CAAP-AF score, provides a reliable prediction of early recurrence risk. However, this approach has certain limitations: (i) The sample size was limited (*n* = 56), with only 13 recurrence events. Given this low event count, multivariable logistic regression and nomogram development are at high risk of overfitting, which may lead to unstable estimates and overly optimistic performance, thereby limiting the model’s robustness and generalizability. Therefore, this nomogram should be considered an exploratory tool, and its potential clinical utility requires confirmation in larger cohorts and through external validation. (ii) Some asymptomatic episodes of AF might have gone undetected during follow-up. Future studies should consider implantable ECG monitoring devices to enhance the detection of early recurrence. (iii) There is currently no consensus on the optimal method for measuring EAT thickness via ultrasound, warranting further research to establish standardized protocols. By addressing these limitations, future studies can enhance the predictive accuracy and clinical applicability of this approach.

## 5 Conclusion

The CAAP-AF score and EAT thickness were associated with early recurrence after catheter ablation in patients with atrial fibrillation, and their combination demonstrated improved discriminatory performance for early recurrence in this cohort. However, given the small sample size and limited number of recurrence events, the combined model and the resulting nomogram should be considered exploratory. Larger prospective, multicenter studies with external validation are warranted to confirm the incremental value of adding EAT thickness, evaluate generalizability across different populations, and establish standardized measurement protocols before routine clinical implementation.

## Data Availability

The raw data supporting the conclusions of this article will be made available by the authors, without undue reservation.

## References

[1] Chung MK Refaat M Shen W Kutyifa V Cha Y Di Biase L Atrial fibrillation: jacc council perspectives. J Am Coll Cardiol. (2020) 75:1689–713. 10.1016/j.jacc.2020.02.025 32273035

[2] Okamatsu H Okumura K Onishi F Yoshimura A Negishi K Tsurugi T Safety and efficacy of ablation index-guided atrial fibrillation ablation in octogenarians. Clin Cardiol. (2023) 46:794–800. 10.1002/clc.24031 37199002 PMC10352973

[3] Tong F Sun Z. Therapeutic effect of his-purkinje system pacing proportion on persistent atrial fibrillation patients with heart failure. Front Cardiovasc Med. (2022) 9:829733. 10.3389/fcvm.2022.829733 35282341 PMC8907546

[4] Lambert C Arderiu G Bejar MT Crespo J Baldellou M Juan-Babot O Stem cells from human cardiac adipose tissue depots show different gene expression and functional capacities. Stem Cell Res Ther. (2019) 10:361. 10.1186/s13287-019-1460-1 31783922 PMC6884762

[5] Zou L Xiao X Jia Y Yin F Zhu J Gao Q Predicting coronary atherosclerosis heart disease with pericoronary adipose tissue attenuation parameters based on dual-layer spectral detector computed tomography: a preliminary exploration. Quant Imaging Med Surg. (2023) 13:2975–88. 10.21037/qims-22-1019 37179933 PMC10167438

[6] Zhao Z Zhang F Ma R Bo L Zhang Z Zhang C Development and validation of a risk nomogram model for predicting recurrence in patients with atrial fibrillation after radiofrequency catheter ablation. Clin Interv Aging. (2022) 17:1405–21. 10.2147/CIA.S376091 36187572 PMC9521706

[7] Winkle RA Jarman JWE Mead RH Engel G Kong MH Fleming W Predicting atrial fibrillation ablation outcome: the caap-af score. Heart Rhythm. (2016) 13:2119–25. 10.1016/j.hrthm.2016.07.018 27435586

[8] Chinese Society of Cardiology, Chinese Medical Association, Heart Rhythm Committee of Chinese Society of Biomedical Engineering. Chinese Guidelines on diagnosis and management of atrial fibrillation. Chinese Journal of Cardiology. (2023) 06:572–618. 10.3760/cma.j.cn112148-20230416-00221 37312479

[9] Joglar JA Chung MK Armbruster AL Benjamin EJ Chyou JY Cronin EM 2023 Acc/aha/accp/hrs guideline for the diagnosis and management of atrial fibrillation: a report of the American College of Cardiology/American Heart Association joint committee on clinical practice guidelines. Circulation. (2024) 149:e1–156. 10.1161/CIR.0000000000001193 38033089 PMC11095842

[10] Mulder MJ Kemme MJB Hopman LHGA Kuşgözoğlu E Gülçiçek H van de Ven PM Comparison of the predictive value of ten risk scores for outcomes of atrial fibrillation patients undergoing radiofrequency pulmonary vein isolation. Int J Cardiol. (2021) 344:103–10. 10.1016/j.ijcard.2021.09.029 34555444

[11] Wen S Liao Y Khurana TS Bai R. Reconsideration of the definition of blanking period and significance of early recurrences after catheter ablation of atrial fibrillation. Curr Opin Cardiol. (2024) 39:15–9. 10.1097/HCO.0000000000001096 37751368

[12] Steinberg C Champagne J Deyell MW Dubuc M Leong-Sit P Calkins H Prevalence and outcome of early recurrence of atrial tachyarrhythmias in the cryoballoon vs irrigated radiofrequency catheter ablation (circa-dose) study. Heart Rhythm. (2021) 18:1463–70. 10.1016/j.hrthm.2021.06.1172 34126269

[13] Jia S Yin Y Mou X Zheng J Li Z Hu T Association between triglyceride-glucose index trajectories and radiofrequency ablation outcomes in patients with stage 3d atrial fibrillation. Cardiovasc Diabetol. (2024) 23:121. 10.1186/s12933-024-02219-w 38581024 PMC10998403

[14] Yang Z Tang L Huang S. Predictive value of caap-af scores and apple scores on recurrence after catheter ablation of patients with atrial fi brillation and heart failure. Chinese Journal of Interventional Cardiology. (2020) 28:430–5.

[15] Sanhoury M Moltrasio M Tundo F Riva S Dello Russo A Casella M Predictors of arrhythmia recurrence after balloon cryoablation of atrial fibrillation: the value of caap-af risk scoring system. Journal of Interventional Cardiac Electrophysiology: An International Journal of Arrhythmias and Pacing. (2017) 49:129–35. 10.1007/s10840-017-0248-4 28417287

[16] Li M Ning Y Tse G Saguner AM Wei M Day JD Atrial cardiomyopathy: from cell to bedside. Esc Heart Fail. (2022) 9:3768–84. 10.1002/ehf2.14089 35920287 PMC9773734

[17] Guldberg E Diederichsen SRZG Haugan KJR Brandes A Graff C Krieger D Epicardial adipose tissue and subclinical incident atrial fibrillation as detected by continuous monitoring: a cardiac magnetic resonance imaging study. Int J Cardiovasc Imaging. (2024) 40:591–9. 10.1007/s10554-023-03029-z 38245893 PMC10951027

[18] Takahashi N Abe I Kira S Ishii Y. Role of epicardial adipose tissue in human atrial fibrillation. J Arrhythm. (2023) 39: 93–110. 10.1002/joa3.12825 37021018 PMC10068928

[19] Feng L Li L Bai L Tang L Zhao Y Zhao X. The association between features of epicardial adipose tissue and the risks of early recurrence after catheter ablation in patients with atrial fibrillation. Front Cardiovasc Med. (2025) 12:1480473. 10.3389/fcvm.2025.1480473 40041172 PMC11877446

[20] Momot K Krauz K Pruc M Szarpak L Rodkiewicz D Mamcarz A. Association between left atrial epicardial adipose tissue attenuation assessed by cardiac computed tomography and atrial fibrillation recurrence following catheter ablation: a systematic review and meta-analysis. J Clin Med. (2025) 14:4771. 10.3390/jcm14134771 40649145 PMC12251091

[21] Momot K Pruc M Rodkiewicz D Ko Luk E Krauz K Pi Tkowska A Predictive value of epicardial adipose tissue parameters measured by cardiac computed tomography for recurrence of atrial fibrillation after pulmonary vein isolation. J Clin Med. (2025) 14:6963. 10.3390/jcm14196963 41096041 PMC12524481

[22] Cohen-Dor S Rav-Acha M Shaheen F Chutko B Labrisch-Kaye H Ben-Haim Z Prediction of atrial fibrillation using radiomic features of left atrial epicardial adipose tissue on noncontrast cardiac computed tomography. Cjc Open. (2025) 7:936–47. 10.1016/j.cjco.2025.03.024 40698311 PMC12277846

