# Supplementary figures and images for: The predictive value of combining CAAP-AF score and epicardial adipose tissue thickness for early recurrence after catheter ablation in atrial fibrillation patients
Source: Front Cardiovasc Med. 2026 May 20;13:1839244. doi: 10.3389/fcvm.2026.1839244 (PMC13229722; doi:10.3389/fcvm.2026.1839244)

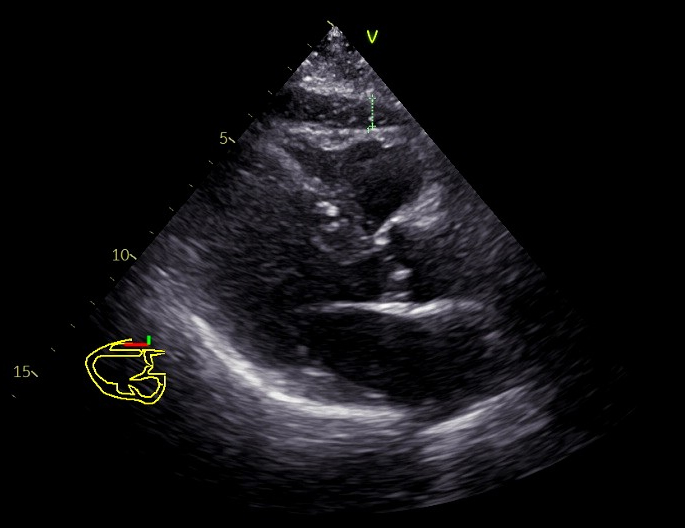

Supplement: Supplementary file 1 [file Image1.jpg]

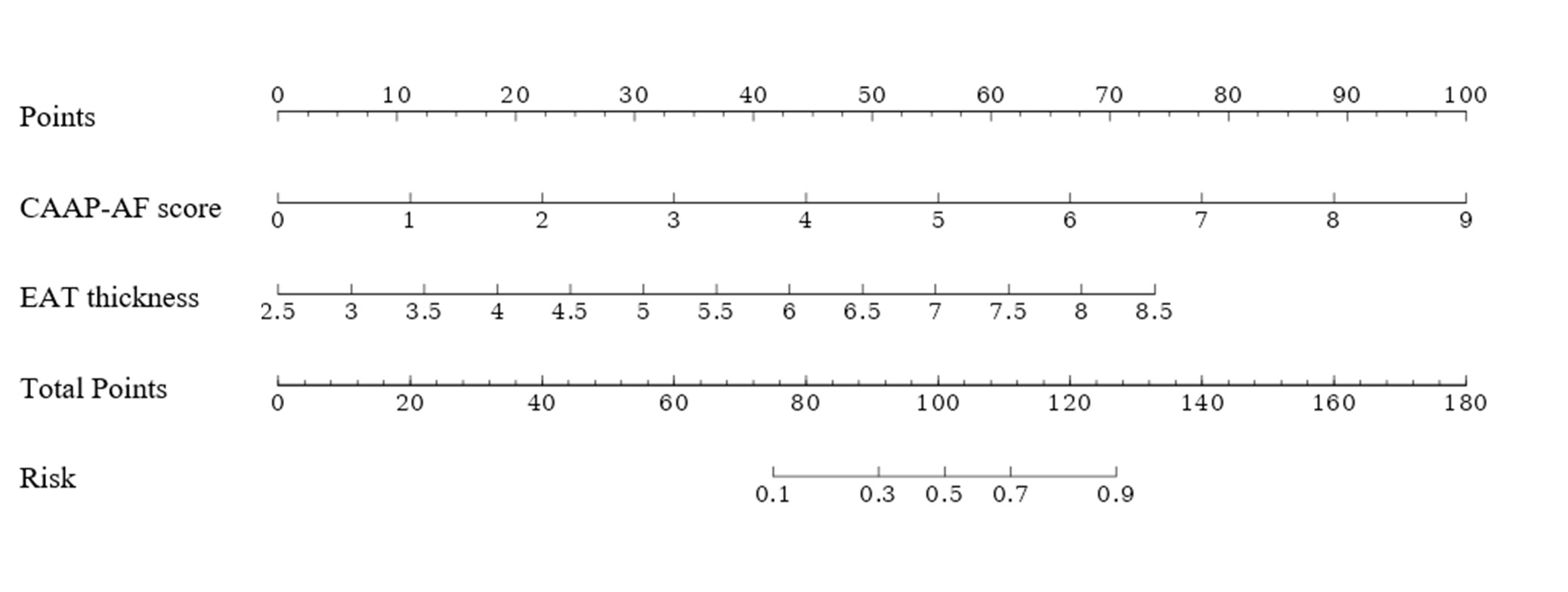

Supplement: Supplementary file 2 [file Image2.jpg]

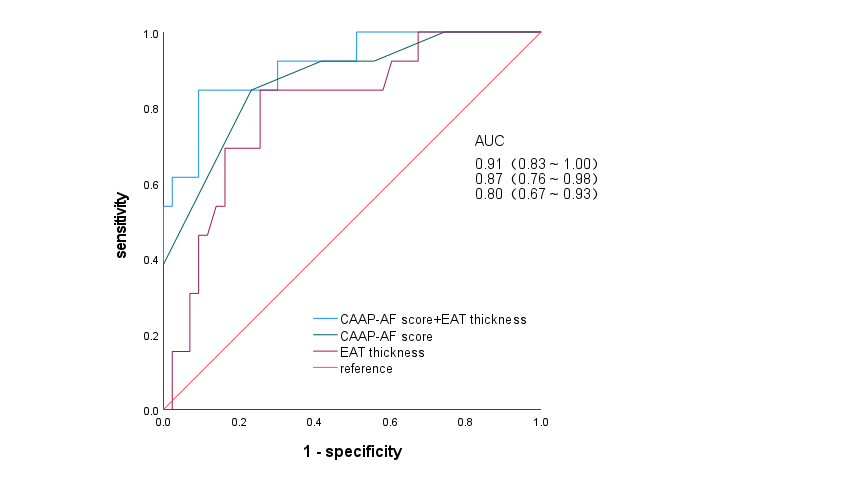

Supplement: Supplementary file 3 [file Image3.jpg]

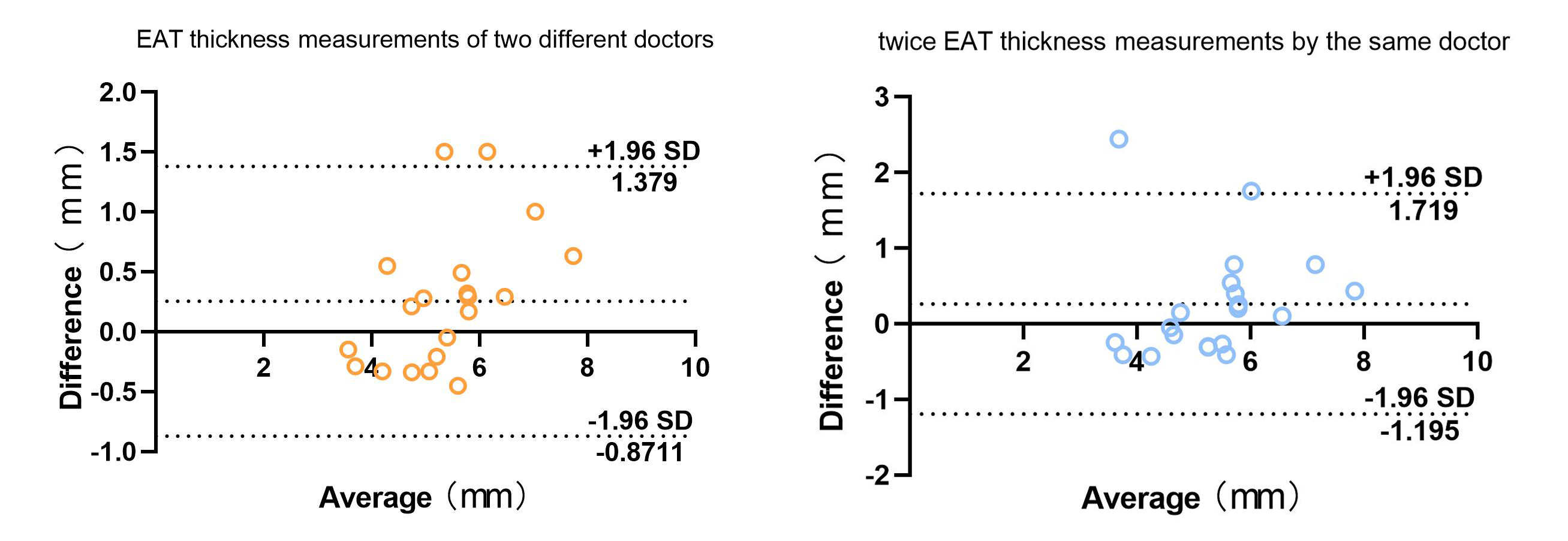

Supplement: Supplementary file 4 [file Image4.jpg]
